# Nursing graduates’ perceived future career pathway and career shift tendency in Egypt: a cross sectional study

**DOI:** 10.1186/s12912-025-02709-6

**Published:** 2025-02-18

**Authors:** Mohamed Hashem Kotp, Mohamed Ahmed Aly, Hossam Aly Ismail, Aliaa Ezz Eldin Abd Elmoaty, Hasan Ahmed Awad Basyouny

**Affiliations:** 1 Nursing Administration Department, Faculty of Nursing, Helwan University, Cairo, Egypt; 2 Department of Nursing, College of Applied Medical Sciences, Prince Sattam Bin Abdulaziz University, Wadi Addwasir, Saudi Arabia

**Keywords:** Nursing graduates, Career pathway, Career shift

## Abstract

**Purpose:**

Addressing the dynamics shaping nurses’ career trajectories and strategies to support workforce stability and retention in the healthcare sector. The aim of this study was to explore nursing graduates’ perceptions of their future Career pathways, and their tendency toward career shifts. Additionally, the study examined the factors influencing career shift tendencies and the relation between career pathway perceptions and career shift tendencies.

**Methods:**

A descriptive cross-sectional study was used, with an online distributed questionnaire for study participants from different nursing graduates’ categories. That included demographic characteristics, a section that measured nursing graduates’ future career pathway perception, and career shift tendency.

**Results:**

Nursing graduates in Egypt were having a positive perception regarding their future career pathway. However, more than one quarter of them were considering career shift represents potential workforce instability and dissatisfaction within the nursing profession and financial factors was one of the main provoking drivers for career shift tendency. In addition, nursing graduates’ perception of career pathway had a significant effect on their career shift tendency in future.

**Conclusion:**

Based on the findings of this study, further efforts should be made to confront nursing shortage in Egypt, implementing targeted interventions to support nursing graduates in navigating their career pathways. Additionally, proactive measures should be taken to address the identified factors driving intentions to leave the profession, such as improving workplace conditions, fostering intergenerational collaboration, and offering mentorship programs. These strategies are essential for fostering a resilient and satisfied nursing workforce and ensuring the delivery of high-quality patient care in the healthcare sector.

**Supplementary Information:**

The online version contains supplementary material available at 10.1186/s12912-025-02709-6.

## Background

The career trajectories of nursing graduates and their propensity for career shifts are critical considerations in understanding the dynamics of the nursing workforce and ensuring the provision of high-quality healthcare services [1, 2]. Nursing, as a profession, offers diverse career pathways ranging from traditional clinical roles to non-traditional positions in education, management, research, and advanced practice nursing. However, recent years have witnessed changes in the aspirations and career preferences of nursing graduates, reflecting broader shifts in the healthcare landscape [3].

Therefore, newly graduate nurses encounter numerous career options, spanning hospital environments and community-cantered primary healthcare services like general practice with various scopes. A key approach to workforce development involves maintaining a steady influx of recent nursing graduates entering employment in diverse clinical settings, especially in regions where shortages are anticipated or prevalent. To support and reinforce the pool of graduate nurse professionals, it becomes imperative to grasp the factors that shape the career decisions of undergraduate students [4].

Career pathway planning is an ongoing process through which an individual sets career goals and identifies the means to achieve them [5]. It is a process of systematically matching career goals and individual capabilities with opportunities for their fulfilment [6]. It’s not a one-time event but is rather a process that becomes part of the repertoire of skills and experiences that enables graduate nursing students to develop as professionals [7].

According to statistics from the American Association of Colleges of Nursing (AACN), the demand for nursing professionals continues to rise, driven by factors such as population aging, increasing chronic disease burden, and healthcare reform initiatives. AACN’s report highlights that nursing schools across the United States are struggling to expand enrolment capacity to meet the growing demand for nurses, resulting in a projected shortage of registered nurses by the year 2030 [1]. This projected shortage underscores the importance of understanding nursing graduates’ career pathways and their potential for career shifts, as these factors can significantly impact the supply of nursing professionals in the workforce. Which could potentially mitigate workforce gaps, particularly by retaining more nurses within the profession. Similar concerns about workforce sustainability are evident globally, including in Egypt, where nursing graduates face significant challenges such as low salaries, poor working conditions, and limited professional development opportunities.

Moreover, studies have indicated a growing trend among nursing graduates to explore alternative career pathways beyond traditional clinical roles. In the United Kingdom many nursing graduates are considering diverse career options, including shifting to other fields such as business and industry [3]. Similarly, in Australia indicated a significant proportion of nursing graduates expressing interest in non-nursing roles or pursuing further education in another specialization [8].

Despite the extensive research on nursing career pathways and job satisfaction globally, there remains a substantial gap in understanding the specific career shift tendencies of nursing graduates, particularly in developing countries like Egypt. While studies have explored nursing workforce trends in Western countries, the Egyptian nursing context—with its unique socio-economic and healthcare challenges—has been largely overlooked. A study by Cottle examines factors influencing early career employment outcomes for nurses, particularly in Western settings. However, her research primarily focuses on nurses’ career settings and intentions to remain in the workforce, which differs from the context of nursing career shifts.

Specifically, there is limited research exploring the specific career shift tendencies and retention challenges faced by nursing graduates in Egypt. The socio-economic, cultural, and healthcare system dynamics in Egypt present unique challenges that are underrepresented in existing studies. For instance, Egyptian nursing graduates often confront a combination of low salaries, limited career progression, and a shortage of qualified mentors, which drive many to seek opportunities abroad or in alternative professions, which may influence career decisions in a very different way. This highlights the need for research that delves into local factors that affect nursing graduates’ career pathways and shifts in regions like Egypt [9].

Similarly, Laschinger et al. study the transition of new graduate nurses into practice, emphasizing their early career satisfaction and adjustment to clinical settings. While this provides valuable insight into the initial career stages, it overlooks how long-term career satisfaction and external factors—such as healthcare system limitations or economic challenges—may influence whether nurses stay within the profession or shift careers. These studies underscore the need for research that looks at career shifts not only during the early transition but throughout a nurse’s entire career pathway [10].

In Egypt, Abd-elmonem et al. explored entrepreneurial tendencies among new graduate nurses and how these relate to their professional development and career aspirations. However, while their study touches upon career aspirations, it does not directly investigate the specific motivations behind career shifts or how graduates perceive their long-term career pathways. This creates a significant gap in understanding the broader picture of how Egyptian nursing graduates view their professional futures and the factors influencing these decisions to leave clinical roles or transition into non-clinical careers [11].

The career aspirations and trajectories of nursing graduates are pivotal considerations in the landscape of healthcare delivery, not only in global contexts but also within specific regions such as Egypt. The nursing profession in Egypt faces unique challenges and opportunities shaped by cultural, economic, and healthcare system factors. Understanding the career pathways and tendencies of nursing graduates in Egypt is crucial for addressing workforce needs, ensuring quality patient care, and advancing the nursing profession in the country [9].

According to Central Agency for Public Mobilization and Statistics [12] total nursing staff who actually work reached 227 thousand nurses in 2020 compared to 229 thousand nurses in 2019, an increase, while the Initial indicators in last 3 years indicates a sharp decrease in healthcare providers specially nurses numbers as result of migration and career shift.

Research specific to Egypt’s nursing workforce landscape is limited but emerging. Studies have shown that Egyptian nursing graduates often encounter challenges in securing employment opportunities that align with their career aspirations [2]. Factors such as limited job prospects, inadequate professional development opportunities, and disparities in salary and benefits influence nurses’ decisions regarding their career pathways and potential shifts within or outside the nursing profession. Despite the increasing demand for nurses in Egypt, few studies have explored the career aspirations and shift tendencies of newly graduated nurses in the region. Understanding the factors influencing these decisions is critical for addressing workforce instability and improving nurse retention, particularly in light of Egypt’s unique healthcare and economic challenges. Moreover, cultural norms and societal perceptions of nursing as a profession may impact nursing graduates’ career choices in Egypt. While nursing is a respected profession, misconceptions about the role and scope of nursing practice persist, affecting career advancement opportunities and job satisfaction among nursing professionals [13].

The migration of nursing graduates from Egypt has been a growing concern in recent years, driven by several key factors. Financial instability, including low wages within the public healthcare sector, plays a central role in this migration trend. Despite the essential role nurses play in healthcare, many nursing graduates find it difficult to sustain themselves and their families due to low salaries and limited opportunities for career advancement in Egypt’s healthcare system. Additionally, the perception of better job prospects and higher salaries abroad, particularly in countries like the Gulf States, the United States, and Canada, motivates many Egyptian nurses to seek employment opportunities overseas. The global demand for healthcare professionals, especially in developed countries, further amplifies this trend. Furthermore, the lack of adequate resources, training opportunities, and career development programs in the domestic healthcare system also contributes to the desire to migrate, as many nurses seek better work environments, job security, and career growth opportunities abroad [14].

While the nursing profession offers a wealth of opportunities for career advancement and professional growth, various factors influence nursing graduates’ career decisions and propensity for career shifts [9]. These factors may include job satisfaction, work-life balance, opportunities for advancement, salary considerations, workplace culture, and external factors such as changes in healthcare policies and technological advancements [15].

Furthermore, the COVID-19 pandemic has brought attention to the resilience and adaptability of nursing professionals. Studies examining the pandemic’s impact on nursing graduates’ career aspirations and shift tendencies can provide valuable insights into how global health crises shape healthcare workforce dynamics [16].

The current study addresses this critical gap by exploring how nursing graduates perceive their career pathways, their tendencies toward career shifts, and the factors influencing these decisions. By doing so, it offers a unique contribution to the literature, providing much-needed insights into the career trajectories of Egyptian nurses. Understanding these factors is crucial for informing policies aimed at improving nurse retention and addressing workforce shortages in Egypt’s healthcare sector. Understanding such issues in Egypt requires nuanced exploration of the factors influencing their career decisions and aspirations. By addressing the barriers to career advancement and providing tailored support and opportunities for professional growth, also ensure a resilient and skilled nursing workforce capable of meeting the evolving healthcare needs of its population.

### Operational definition

#### Nursing graduates

Refers to nurses who have between 1 to 5 years or more of clinical experience post-graduation. This ensures that participants have sufficient work experience to provide relevant insights while still being early in their careers. This operational definition is combining of and consistent with definitions of “graduate nurses” and “early-career nurses” commonly used in global nursing literature and professional standards [14, 17–19].

### Purpose of study

The aim of this study was to explore perceived future Career pathway, as well as career shift tendency among nursing graduates in Egypt. This study also investigated factors influencing career shift tendency. And examine the relation between career pathway perception and career shift tendency.

## Methods

### Design

This research was conducted as a cross-sectional study, from different nursing graduates’ categories (technical institute, bachelor’s degree, postgraduate degrees practicing in clinical settings (bed side nurses, nurse educators, and nurse leaders), academic settings. The study was conducted from March 2023 to December 2023. The quantitative approach allowed for the assessment of patterns, relationships, and statistical significance between variables, providing a comprehensive understanding of the factors influencing career decisions within the nursing profession. This design aligns directly with the study’s objectives, using data that can be statistically analyzed to draw conclusions about the workforce dynamics among nursing graduates.

### Participants

In this study a convenience sampling method was employed to survey nurse graduates from different categories, Egypt. The inclusion criteria for this study were as follows: (a) nurses with a minimum of one year of clinical experience, (b) registered nurses, (c) nurse leaders and (d) voluntary participation in the study.

The non-entry criteria were as follows: (a) nursing interns or trainees, (b) nurses who were not on duty during the survey period, and (c) nurses who declined to participate in the study. to focus on individuals who had completed their internships and had gained some level of professional independence.

Ultimately, the research encompassed 1000 nurses out of a target population of 218 thousand nurses according to Central Agency for Public Mobilisation and Statistics, Egypt (2021).

For this study, newly graduated nurses are defined as individuals who have completed their nursing degree and possess 1 to 5 years of clinical experience. This definition excludes nurses with less than one year of experience to ensure that participants have a substantive professional background.

The sample size was calculated using G Power to detect a medium effect size (f2 = 0.15) with a power of 0.80 and a significance level of 0.05. Based on the target population of 218,000 nurses, the calculation suggested a sample size of 1,000 participants to ensure adequate statistical power.

### Data collection

The data collection process for this study employed online method. Google form sheet questionnaire was distributed for participants in various hospitals, universities institutes and medical centres via phone, email, and other means to extend invitations to participate in the research.

The inclusion of both staff nurses and nurse leaders was intended to capture a range of career pathways. While we acknowledge the potential for bias, this approach was chosen to explore different career trajectories within the nursing profession. To control for bias, statistical adjustments (such as multivariate analysis) were used to account for differences in employment status and experience levels.

Participants were recruited through an online questionnaire distributed via email, professional nursing associations, and social media. Invitations were extended to various hospitals, universities, and medical centers to ensure voluntary participation across different categories of nurses.

Subsequently, the distributed questionnaire form included prefilling instructions to ensure a clear understanding of the research objectives and content. Emphasis was placed on respecting the rights of participating nurses and underscoring the significance of obtaining informed consent as an item at the beginning of questionnaire indicating accepting to participate in the study and required to answer before completing and submitting the response. During the data collection phase, researchers provided detailed explanations to participating nurses regarding the study’s purpose, expected outcomes, and the importance of their involvement. It was explicitly highlighted that participation in this study was entirely voluntary, and participants had the option to refuse participation. Additionally, the research’s confidentiality and data protection measures were clearly articulated, ensuring full respect for participants’ privacy.

Missing data and outliers were addressed through standard data cleaning procedures. For missing data, cases with incomplete responses were handled using a combination of techniques, including listwise deletion for participants with significant missing information, and imputation for missing values in specific variables using mean substitution, where appropriate, to maintain sample size and minimize bias.

### Pilot study

A pilot study was conducted with 100 participants representing the target population of graduated nurses. Feedback from this pilot informed adjustments to ensure clarity, relevance, appropriateness and coverage of all dimensions related to career pathways and shift tendencies. Such as refining the wording of certain survey items and we expanded on certain areas, such as work-life balance and financial considerations, which were highlighted by pilot participants as critical factors influencing career decisions. Adding suggested relevant factors influencing career to ensure greater clarity and ease of understanding. Construct validity was assessed using Exploratory Factor Analysis (EFA), with Bartlett’s test of sphericity yielding a significant result (*p*. < 0.001) and the KMO measure of sampling adequacy surpassing the acceptable value.

### Instruments

#### Demographic characteristics

The demographic characteristics included gender, age, highest nursing degree earned, years since graduation (experience), current employment status, current work setting.

#### Future career pathway perception and career shift tendency questionnaire

The initially developed by the investigators, it consists of two parts. First part was consisted of four distinct dimensions with 21 items, these dimensions included: Immediate career goals (comprising 5 items), motives to explore current career pathways (containing 4 items), Challenges or obstacles pursuing chosen career pathway (containing 4 items), Long-term career goals as a nursing graduate (comprising 8 items). Second part was consisted of 2 dimensions with 11 items, these dimensions included: Considering intention for career shift (comprising 1 item), Factors influencing consideration of a career shift (comprising 10 items), and considered field for a career shift (comprising 1 item). Respondents utilize a 3-point Likert scoring system, with response options ranging from "Disagree" to "Agree," correspondingly scored from 1 to 3. The cumulative score on this scale falls within the range of 32 to 96 points, with higher scores denoting a more robust perception of career pathway. It is noteworthy that a Cronbach’s α coefficient of 0.791 in this study. Second part was used to assess nursing graduates’ career shift tendency, it was consisted of four items.

*Career Pathway Perception*: refers to the way in which nursing graduates view their current and future career prospects within the nursing profession. It encompasses their attitudes toward career development opportunities, job satisfaction, and long-term goals within nursing, including aspirations for further education, specialization, and career advancement.

*Career Shift Tendency*: describes the inclination or intention of nursing graduates to consider a career change, either within the healthcare sector or outside of it, based on various influencing factors such as job satisfaction, financial motivations, work-life balance, or external opportunities (Table 1).

**Table 1:** Future career pathway perception and career shift tendency questionnaire description

Construct	Dimensions	No of items	Scoring
**Career Pathway Perception**	Immediate Career Goals	5 items	3-point Likert scale: 1 = Disagree, 2 = Neutral, 3 = Agree
	Motives to Explore Career Pathways	4 items	
	Challenges in Career Pathway	4 items	
	Long-term Career Goals	8 items	
**Career Shift Tendency**	Intention for Career Shift	1 item	3-point Likert scale: 1 = Disagree, 2 = Neutral, 3 = Agree
	Factors Influencing Career Shift	10 items	
	Field for Career Shift Consideration	1 item	

### Measurements

#### Exploratory Factor Analysis (EFA)

Bartlett’s test of sphericity was performed to determine the adequacy of the data for factor analysis, yielding a significant result (*p* < 0.001). Additionally, the Kaiser–Meyer–Olkin (KMO) measure of sampling adequacy was calculated and surpassed the minimum acceptable value of 0.7, confirming the suitability of the dataset for factor analysis. A principal component analysis (PCA) with varimax rotation was conducted on the data from the future career pathway perception and career shift tendencies questionnaire (32 items), as mentioned in (Tables 2, 3).

**Table 2:** Reliability analysis (internal consistency and test–retest reliability)

Scale	Cronbach’s Alpha	Test–Retest Reliability (Spearman)
Nurse graduates’ career pathway perception (Overall)	0.90	0.834
- Career goals (Factor 1)	0.90	
- Motivators (Factor 2)	0.88	
- Challenges (Factor 3)	0.87	
Career shift tendency (Overall)	0.88	0.835
- Intention of career shift (Factor 1)	0.87	
- Anticipated factors in considered career shift (Factor 2)	0.86

**Table 3:** Exploratory Factor Analysis (EFA) results for nursing graduates’ career pathway perception

Item Description	Factor 1: Career goals	Factor 2: Staff Motivators	Factor 3: Challenges	Factor Loadings
I Plan for advancement in the current nursing role (e.g., managerial roles)	0.781	-	-	0.781
I Pursue a specialized nursing certification (e.g., Critical Care, Oncology)	0.794	-	-	0.794
I Pursue advanced nursing education (e.g., MSN, DNP)	0.816	-	-	0.816
I Plan for transition to a different nursing specialty	0.760	-	-	0.760
Plan for transition to a non-nursing healthcare role	0.783	-	-	0.783
Passion for the field	-	0.824	-	0.824
Opportunities for advancement	-	0.793	-	0.793
Desire to make a difference	-	0.813	-	0.813
Financial prospects	-	0.799	-	0.799
Lack of financial resources	-	-	0.842	0.842
Limited time for additional education	-	-	0.825	0.825
Job market competitiveness	-	-	0.806	0.806
Family commitments	-	-	0.790	0.790
Nursing leadership/management roles	0.780			0.780
Nurse educator/teaching roles	0.764			0.764
Academic roles	0.734			0.734
Nursing informatics role	0.745			0.745
Healthcare quality role	0.758			0.758
Nurse researcher/clinical research	0.782			0.782
Advanced nursing practice (e.g., Nurse Practitioner)	0.732			0.732
Transition to non-healthcare career	0.752			0.752

The exploratory factor analysis (EFA) of the future career pathway perception and career shift tendencies questionnaire identified three common factors, explaining a significant portion of the total variance. All 32 items loaded significantly onto these five factors, with factor loadings ranging from 0.760 to 0.842.

- Factor 1 was labeled “Career Goals,” capturing items related to advancement in nursing profession, certification, education.
- Factor 2 was labeled “Motivators,” reflecting passion, opportunities, making a difference, financial prospects.
- Factor 3 was termed “Challenges,” focusing on lack of financial resources, time, job market competitiveness.
- Factor 4 was termed “Intention for career Shift,” focusing on considering career shift within months.
- Factor 5 was termed “Anticipated factors in considered career shift,” focusing on field of the career shift and Challenges or obstacles anticipated in pursuing chosen career pathway.

The exploratory factor analysis (EFA) of the career pathway questionnaire identified two common factors “Career pathway perception and Career Shift Tendency” explaining a significant portion of the total variance. All 32 items loaded significantly onto these two factors, with factor loadings ranging from 0.756 to 0.836.

Together, these factors reflect the multifaceted graduates perceived future career goals and their tendency to shift their career, moreover their anticipated Challenges or obstacles in pursuing chosen career pathway.

#### Confirmatory Factor Analysis (CFA)

Confirmatory Factor Analysis (CFA) was performed on a sample of 1000 nurses to validate the structure of the questionnaire: nursing graduates perceived future Career pathway part (three-factor model) and the career shift tendency part (two-factor model). The results indicated that the three-factor model for the Nurse Leaders’ Readiness survey had a good fit with the data, as evidenced by the following fit indices: χ2/df = 2.677, CFI = 0.809, GFI = 0.855, AGFI = 0.802, RMSEA = 0.053, and RMR = 0.051. These indices demonstrate a robust model fit, with values aligning closely with the ideal standards for CFI, GFI, and RMSEA.

Similarly, the two-factor model for the career shift tendency showed an acceptable fit, with fit indices of χ2/df = 2.008, CFI = 0.903, GFI = 0.800, AGFI = 0.812, RMSEA = 0.063, and RMR = 0.060. The high CFI and GFI values, coupled with low RMSEA and RMR, confirm the validity of this model in evaluating career shift tendency, particularly in areas related to Factors influencing consideration of a career shift, and Challenges or obstacles anticipated in pursuing chosen career pathway. Overall, the CFA results endorse the reliability and validity of both models, indicating they effectively measure the intended constructs, see more in Table 4.

**Table 4:** Confirmatory factor analysis

Fit Indices	Nurse graduates’ future career pathway perception (Three-Factor Model)	Career shift tendency (Two-Factor Model)
χ^2^/df	2.677	2.008
Comparative Fit Index (CFI)	0.809	0.903
Goodness of Fit Index (GFI)	0.855	0.800
Adjusted GFI (AGFI)	0.802	0.812
RMSEA	0.053	0.063
RMR	0.051	0.060

### Reliability analysis

The internal consistency of the scales was assessed using Cronbach’s alpha, with the Nurse graduates’ future career pathway perception questionnaire showing an overall Cronbach’s alpha of 0.90, indicating high internal consistency. Within this questionnaire, Factor 1 (Career Goals) had a Cronbach’s alpha of 0.90, Factor 2 (Motivators) had an alpha of 0.88, and Factor 3 (Challenges) exhibited an alpha of 0.87. The career shift tendency demonstrated a Cronbach’s alpha of 0.88, confirming strong internal reliability, with Factor 1 (Intention of career shift) showing a Cronbach’s alpha of 0.87 and Factor 2 (Anticipated factors in considered career shift) having an alpha of 0.86. To evaluate test–retest reliability, a random subset of 100 nurses completed the surveys again two weeks after the initial administration, with the Future Career Pathway Perception and Career Shift Tendency questionnaire achieving a Spearman correlation coefficient of 0.834 and the Perceived career pathway perception achieving a Spearman correlation of 0.835, both surpassing the 0.7 threshold for reliability. These results confirm that the questionnaire is a reliable tool for assessing nursing graduates’ perception of their career pathway and career shift tendency (Table 5).

**Table 5:** Exploratory Factor Analysis (EFA) results for career shift tendency

Item Description	Factor 1: Intention of career shift	Factor 2: Anticipated factors	Factor Loadings
I am considering shifting my career within months	0.803	-	0.803
Desire for a Change	-	0.837	0.837
Better Career Opportunities	-	0.794	0.794
Work-Life Balance	-	0.822	0.822
Financial Growth	-	0.810	0.810
International Experience	-	0.757	0.757
Lack of financial resources	-	0.824	0.824
Limited time for additional education	-	0.793	0.793
Job market competitiveness	-	0.813	0.813
Family commitments	-	0.799	0.799
Considered field for career shift	-	0.737	0.737

### Data analysis

The statistical analysis was conducted using IBM SPSS Statistics, version 26.0. Categorical variables were described in terms of frequencies, percentages, and Mean ± SD for determining perception for study subjects’ career pathway, and their level of career shift tendency. For univariate analysis, the appropriate statistical test was selected based on the data type. To explore the relationships between career pathway perception, and career shift tendency, Pearson’s correlation analysis was used. Variables with *p* values less than.05 in both univariate and correlation analyses were subsequently included in the multiple linear regression analysis to We also performed regression analysis to explore the impact of various demographic and educational variables on career shift tendency, with the model explaining of the variance. Key predictors included age, years of experience, and highest nursing degree earned, with results will indicate the statistical significance. All analyses were conducted using, ensuring data preparation and variable coding were appropriately handled. A significance level of *p* < 0.05 was deemed statistically significant.

### Ethical considerations

Ethics approval was obtained from the Ethical Committee of the Faculty of Nursing at Helwan University with No. (40) 18/3/2023. online google form was shared with study subjects to explain the purpose of the study, clarify the items of the questionnaire, and obtain consent. They were informed about the privacy of their information and were assured that the information would be used for scientific research only. The study was voluntary and harmless. they had the right to refuse to participate in the study or withdraw at any time. No identifying information was collected from them. The researchers explained in the form that the results of this study could influence how nurses and graduates in Egypt perceive their career pathway and give indicators for stakeholders a notion about career shift tendency among nurses and reasons evolving it. Data collected from subjects were stored in a cloud and local drive in the office of the principal researcher.

## Results

Table 6 sociodemographic characteristics illustrates the relationship between nurses’ profiles, future career pathway perceptions, and career shift tendencies among studied nurses. Gender differences are apparent, with male nurses showing a lower mean perception of future career pathways (25.27 ± 5.19) compared to female counterparts (32.79 ± 7.80), and these differences are statistically significant (*p* = 0.001). Age categories also play a role, influencing both future career pathway perception and career shift tendencies. The highest nursing degree earned is associated with variations in perceptions, notably with nurses holding a Bachelor of Science in Nursing (BSN) degree showing higher means for both future career pathways (31.04 ± 10.92) and career shift tendency (15.00 ± 8.92). Additionally, years since graduation reveal nuanced patterns, demonstrating significant variations in both career pathway perception and career shift tendency among nurses with different levels of experience. Furthermore, the current employment status and work setting significantly impact nurses’ career perceptions and shift tendencies.

**Table 6:** Sociodemographic characteristics of the sample (*N* = 1000)

Sociodemographic Characteristics	N	(%)	Future Career Pathway perception, mean ± SD	P	Career Shift Tendency, mean ± SD	P
**Gender**						
Male	300	30.0	25.27 ± 5.19	0.214	9.09 ± 2.30	0.101
Female	700	70.0	32.79 ± 7.80		13.30 ± 4.11	
**Total**	1000	100	58.03 ± 13.11		19.07 ± 6.01	
**Age**						
Less than 25 years	200	15.0	15.05 ± 4.61	.048*	6.09 ± 2.30	.022*
25: 30 years	300	30.0	19.27 ± 6.43		7.13 ± 4.09	
31–35 years	250	25.0	24.84 ± 8.19		9.76 ± 6.22	
36- 40 years	200	20.0	17.57 ± 5.06		8.63 ± 2.67	
More than 40 years	50	5.0	14.00 ± 4.33		6.11 ± 4.23	
**Highest Nursing Degree Earned**						
Diplome	100	10.0	15.10 ± 6.53	.007*	7.51 ± 6.14	.059*
Associate degree	200	20.0	17.97 ± 5.66		9.06 ± 6.02	
Bachelor of Science in Nursing (BSN)	400	40.0	31.04 ± 10.92		15.00 ± 8.92	
Master of Science in Nursing (MSN)	150	15.0	14.55 ± 6.01		8.01 ± 8.94	
Diploma of Nursing Practice (DNP)	80	8.0	9.10 ± 4.03		6.41 ± 5.04	
Nursing PhD	70	7.0	8.70 ± 3.22		4.81 ± 5.64	
**Years Since Graduation (Experience)**						
1–5 years	360	36.0	24.84 ± 8.19	** < 0.001**	16.77 ± 9.91	** < 0.001**
6–10 years	230	23.0	17.97 ± 5.66		13.06 ± 9.02	
11–15 years	180	18.0	15.05 ± 4.61		9.16 ± 8.32	
Over 15 years	80	8.0	9.10 ± 4.03		6.41 ± 5.04	
**Current Employment Status**						
Employed as a bed side nurse	400	40.0	32.79 ± 7.80	.000*	19.79 ± 7.80	.0190*
Employed as nurse manager	150	15.0	17.04 ± 4.60		17.04 ± 4.60	
Healthcare quality specialist nurse	80	8.0	9.69 ± 3.00		9.69 ± 3.00	
Employed in a non-nursing role	120	12.0	16.62 ± 4.11		11.42 ± 4.81	
Academic educational role	140	14.0	18.19 ± 5.43		8.17 ± 5.03	
Case manager	60	6.0	6.01 ± 4.70		5.91 ± 3.60	
Free lancer nurse	40	4.0	2.60 ± 3.51		2.00 ± 1.01	
Nursing science researcher	100	10.0	11.63 ± 5.50		6.04 ± 6.10	
Nursing informatics specialist	60	6.0	6.06 ± 4.83		2.01 ± 2.07	
**Current Work Setting**						
Governmental Hospital	360	36.0	30.49 ± 8.01	.000*	16.09 ± 7.01	** < 0.001**
Private sector hospital	240	24.0	32.00 ± 9.33		14.32 ± 5.21	
Primary healthcare facility	160	16.0	17.14 ± 4.61		11.04 ± 4.60	
University faculty	120	12.0	13.99 ± 4.23		10.01 ± 3.70	
Own business nursing facility	80	8.0	10.01 ± 6.50		9.69 ± 3.10	

*p-value <0.05 is significant

**Fig. 1:**
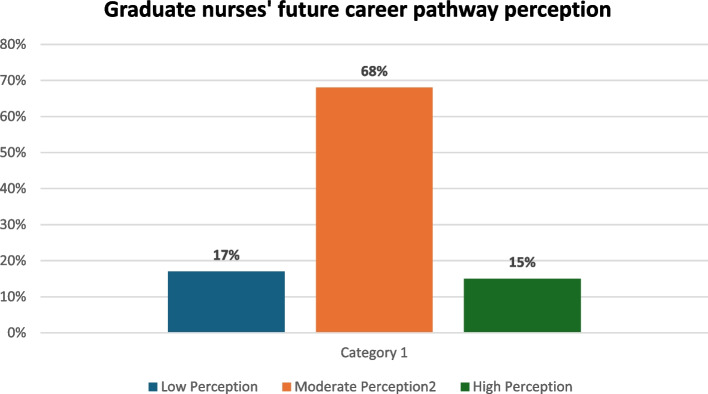
Representing graduate nurses perception level regarding their future career pathway, revealing 68% of them had a moderate level

Table 7 demonstrated Future Career Pathway Perception Notably, 49.3% stated that they wanted to pursue advanced nursing education, while 29.2% aim for advancement in their current nursing roles, showcasing a commitment to professional development. Passion for the field is a significant motivator, with 34.0% citing it as a driving factor, and financial prospects (46.5%) emerge as a key motivator as well. Challenges such as a lack of financial resources (60.9%) and job market competitiveness (31.4%) are acknowledged, emphasizing the importance of addressing these barriers. In terms of long-term goals, academic roles (39.7%) and nursing leadership/management roles (18.6%) are prominent, reflecting a desire for academic and managerial responsibilities. However, it is notable that certain roles, such as nursing informatics, nurse researcher, and advanced practice nursing, currently have zero representation among respondents. The overall mean future career pathway perception is 58.03 ± 13.11, indicating a positive and diverse range of career aspirations and challenges within the nursing profession (Fig. 1).

**Table 7:** Descriptive statistics for future career pathway perception (*N* = 1000)

Future Career Pathway perception	N	(%)	Mean ± SD
**Immediate career goals**			
Advancement in the current nursing role	292	29.2	43.60 ± 8.02
Pursue a specialized nursing certification	45	4.5	
Pursue advanced nursing education (e.g., MSN, DNP)	493	49.3	
Transition to a different nursing specialty	20	20.0	
Transition to a non-nursing healthcare role	150	15.0	
**Motivators to explore pathways**			
Passion for the field	34	34.0	29.71 ± 7.52
Opportunities for advancement	301	30.1	
Desire to make a difference	200	20.0	
Financial prospects	465	46.5	
**Challenges or obstacles anticipated in pursuing chosen career pathway**			
Lack of financial resources	609	60.9	13.44 ± 4.36
Limited time for additional education	53	5.3	
Job market competitiveness	314	31.4	
Family commitments	24	2.4	
**Long-term career goals as a nursing graduate**			
Nursing leadership/management roles	186	18.6	38.09 ± 6.00
Nurse educator/teaching roles	207	20.7	
Academic roles	397	39.7	
Nursing informatics role	0	0	
Healthcare quality role	86	8.6	
Nurse researcher/clinical research	0	0	
Advanced practice nursing (e.g., Nurse Practitioner)	0	0	
Transition to non-healthcare career	124	12.4	
**Total**			**58.03 ± 13.11**

**Fig. 2:**
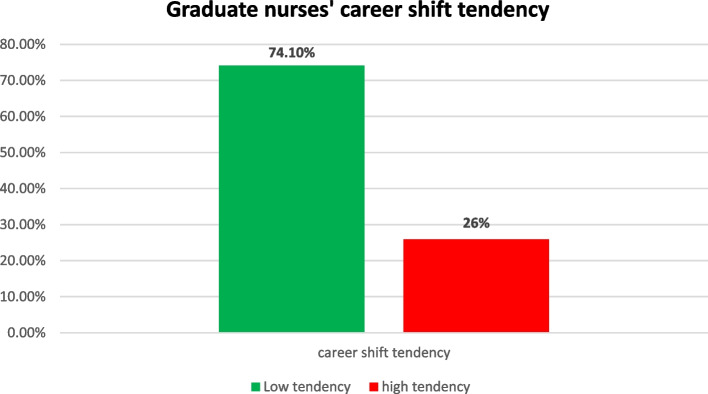
Illustrating graduate nurses career shift tendency, revealing 26% of them had a high tendency level to shift their career

The Table 8 presents insightful information on career shift tendencies among 1000 respondents, revealing that 25.9% are actively considering a change. As regards factors influencing this consideration include a strong desire for change (56.0%), better career opportunities (30.1%), and a focus on work-life balance (74.0%) and financial growth (80.4%). Respondents anticipate challenges, particularly related to financial resources and time constraints for additional education. Notably, fields such as software development (21.0%) and freelancing (27.9%) are prominent considerations for a career shift. The mean values for certain aspects, such as the age-related value for those considering a career shift (12.25 ± 5.12), may warrant further clarification. Overall, the data highlights the dynamic factors influencing career decisions and sheds light on the diverse fields attracting career-shift considerations (Fig. 2).

**Table 8:** Descriptive statistics for career shift tendency (*N* = 1000)

Career shift tendency	N	(%)	Mean ± SD
**Considering career shift**			
Yes	259	25.9	12.25 ± 5.12
No	741	74.1	
**Factors influencing consideration of a career shift**			
Desire for a Change	560	56.0	10.90 ± 4.04
Better Career Opportunities	301	30.1	
Work-Life Balance	740	74.0	
Financial Growth	804	80.4	
International Experience			
**Challenges or obstacles anticipated in pursuing chosen career pathway**			
Lack of financial resources	609	60.9	9.04 ± 3.01
Limited time for additional education	530	53.0	
Job market competitiveness	314	31.4	
Family commitments	240	24,0	
**Field or specialization considered for a career shift**			
Software Developer	210	21.0	15.88 ± 2.57
Social media work	274	27.4	
Telesales	105	10.5	
Marketing management	49	49.0	
Freelancing	279	27.9	
Real Estate Development	36	36.0	
Tourism	21	21.0	
Trading	26	26.0

Table 9 demonstrates a statistically significant positive correlation between future career pathway perception and career shift tendency.

**Table 9:** Relation of career pathway perception and career shift tendency (*N* = 1000)

Variables	1	2
**Future Career Pathway perception**	1	0.449**
**Career shift tendency**	0.663**	2

^**^p < 0.001; 1 = career pathway perception; 2 = career shift tendency

Table 10 shows Pearson’s correlation analysis examining the associations among career pathway perception and career shift tendency are presented in Table 9. The variables accounted for 20.88% of the variance in career shift tendency. It was observed that career pathway perception demonstrates a positive correlation with career shift tendency.

**Table 10:** Regression (Pearson’s correlation) analysis examining the associations among career pathway perception and career shift tendency

Variables	*β*	*SE*	*T*	*P*
**Age (**< 25 **vs.** > 25**)**	**12.030**	**2.024**	**4.090**	** < 0.001**
**Age (**26 ~ 30 **vs.** > 35**)**	**1.733**	**2.043**	**3.012**	**0.091**
**Age (**35 ~ 40 **vs.** > 45**)**	**1.846**	**1.722**	**2.088**	**0.297**
**Years of Experience (**1 ~ 5 **vs.** 6 ~ 10**)**	**7.995**	**3.102**	**4.656**	** < 0.001**
**Years of Working (**6 ~ 10 **vs.**11 ~ 15**)**	**0.068**	**1.016**	**2.778**	**0.052**
**Years of Working (**11 ~ 15 **vs.** > 15**)**	**0.713**	**2.814**	**3.329**	**0.012**
**Highest Nursing Degree Earned** (Technical nurses **vs.** Associate degree nurse)	**4.908**	**2.002**	**2.030**	**0.004**
**Highest Nursing Degree Earned** (Bachelor’s degree nurses **vs.** Master’s degree nurse)	**2.328**	**1.477**	**4.511**	**0.111**
**Highest Nursing Degree Earned** (Diploma of Nursing Practice **vs.** Nursing PhD. nurse)	**1.064**	**2.622**	**0.801**	**0.085**
**Current employment status (**Bed side nurse **vs.** Nurse manager**)**	**1.128**	**1.420**	**1.003**	**0.027**
**Current employment status (**quality specialist nurse **vs.** a non-nursing role**)**	**3.140**	**1.433**	**2.220**	**0.027**
**Current employment status (**Academic educational role **vs.** Case manager**)**	**4.126**	**1.006**	**3.927**	** < 0.001**
**Current employment status (**Free lancer nurse **vs.** Nursing science researcher**)**	**4.064**	**1.622**	**2.110**	**0.027**
**R**^**2**^	**0.174**			
**Adjusted R**^**2**^	**0.143**			

## Discussion

The present study aims to explore nursing graduates perceived future Career pathway and their Career shift tendency. Understanding graduates’ perception toward their career pathway is essential as it influences their decisions regarding further education, specialization, and employment opportunities. Additionally, insights into their career shift tendencies provide valuable information for educational institutions, healthcare organizations, and policymakers to tailor support systems and resources to meet evolving workforce needs.

By comprehensively examining these aspects, stakeholders can foster an environment that facilitates career progression, professional satisfaction, and ultimately, enhances the quality of patient care. As nursing manpower has a considerable shortage, it is necessary for undergraduate nursing students to develop positive attitudes based on logical knowledge about nursing profession. Therefore, the present study explored issues concerning factors affecting how nursing graduates perceive and engage with the profession, aiming to identify key factors that influence their attitudes and decision-making processes.

By understanding these factors, educators and stakeholders can develop targeted interventions and educational strategies to foster positive attitudes toward the nursing profession among undergraduate students. This, in turn, can help mitigate the shortage of nursing manpower by attracting and retaining more individuals in the field, ultimately contributing to the stability and sustainability of the healthcare workforce. The information obtained through this part of the study showed whether there was a need for nursing graduates to be oriented to their career pathway.

This study examined the perceived future career pathway and career shift tendency among nursing graduates. They reported how satisfied they were with their career indicating a positive and diverse range of career aspirations and challenges within the nursing profession. Many graduates in this study reported that they were having proper, positive perception regarding their career pathway; however, a considerable percentage of them reported tendency for career shift. In addition, we found that graduates’ perception of career pathway had a significant effect on their career shift tendency in future.

In the present study, nearly half of the nursing graduates expressed a desire to pursue advanced nursing education as an immediate career goal. This preference is likely driven by the belief that further education can improve job satisfaction and financial rewards. Acquiring specialized knowledge and skills may open up more career opportunities, including higher-paying positions with greater job security. The desire for advanced education reflects a commitment to lifelong learning and a dedication to advancing both individual careers and the nursing profession as a whole.

This finding is consistent with another study [9], which found that most students considered further education as a key goal. Similarly, a previous study [12] reported that 70% of Norwegian nursing students intended to pursue additional education, seeing it as a professional step that would enhance their economic and career prospects. The motivation for advanced education may also stem from the recognition that healthcare is constantly evolving. Continuous learning is essential to provide high-quality care, adapt to new technologies and evidence-based practices, and address emerging health challenges. By investing in further education, nursing graduates may feel more prepared to navigate these changes effectively.

The present study revealed that nearly a half of study subjects reported financial prospects as a motivator to explore pathways. Moreover, as regards challenges or obstacles anticipated in pursuing chosen career pathway, nearly two thirds of nursing graduates highlighted Lack of financial resources as the main obstacle. These results are consistent with previous research [20] who have highlighted the significant influence of financial factors on nursing career decisions, they examined the factors influencing nurse labor market outcomes in the United States and found that salary considerations were among the key determinants affecting nurses’ job choices and career trajectories. Similarly, [21] explored the factors influencing nurses’ intention to leave their current positions in Taiwan and identified salary as one of the main predictors of turnover intention.

In Egypt, as in many other countries, nurses often face challenges such as low salaries, limited opportunities for career advancement, and a lack of adequate resources in healthcare settings. These difficulties can significantly affect their career decisions, with financial considerations playing a central role. Nurses may view pursuing specific career pathways or further education as ways to enhance their financial stability and improve their quality of life. The rising cost of living, inflation, and other economic pressures in Egypt can further heighten the importance of financial security when nursing professionals are making career choices. In this socio-economic environment, many nurses prioritize career paths that not only provide personal satisfaction but also offer financial security and opportunities for upward mobility.

Nurses in Egypt face considerable financial challenges due to relatively low wages in the public healthcare sector, especially in comparison to the private sector or alternative career paths. Economic pressures such as inflation and low salaries significantly affect nurses’ job satisfaction and their long-term commitment to the profession. Financial instability within the healthcare system, combined with limited career progression opportunities in public institutions, often leads nurses to seek better-paying roles either in the private sector or abroad. This trend is further intensified by the mismatch between the qualifications of nurses and the available roles within the healthcare system, creating frustration and prompting many to consider career shifts or migration for improved financial prospects [14].

The socio-economic context and healthcare system dynamics in Egypt significantly influence the career decisions of nursing professionals. Nurses in Egypt face low wages, particularly in the public healthcare sector, which often fails to meet the increasing cost of living. For example, the average salary for nurses in Egypt remains below the national median income, with many nurses earning around 5,500 EGP per month, far from sufficient to cover the rising costs of daily living, such as transportation, housing, and healthcare [22]. As a result, financial pressures have become a key driver of career shifts. Nurses often seek higher-paying opportunities either in the private healthcare sector, where salaries are relatively higher, or in non-medical fields like administration or business [23].

Moreover, systemic challenges in the healthcare sector, such as severe staffing shortages, inadequate resources, and high nurse-to-patient ratios, exacerbate these financial and emotional pressures. A report from the Egyptian Ministry of Health (2021) [24], highlighted that there is a chronic shortage of nursing staff, with a ratio of just 1 nurse for every 10 patients in certain public hospitals, leading to burnout and job dissatisfaction. These factors, combined with limited career advancement opportunities, often push nurses to consider alternative career paths, including migration abroad for better pay and working conditions [25].

The poor working conditions within the Egyptian healthcare system are another key factor influencing career shifts. Chronic staffing shortages, inadequate resources, and high patient-to-nurse ratios contribute to nurse burnout and stress. These systemic challenges not only affect job satisfaction but also increase the likelihood of nurses seeking alternative career paths. Faced with these pressures, nurses may consider shifting within the healthcare sector, such as transitioning from clinical roles to educational or managerial positions, or even leaving the healthcare field entirely for less demanding careers in administration or other industries.

Social factors also have a significant influence on career decisions. In Egypt, cultural expectations related to work-life balance and family responsibilities can be powerful motivators for career shifts, particularly among women. Traditional gender roles often place added pressure on female nurses to prioritize family obligations over professional development, which may lead them to seek more flexible or stable career opportunities. As a result, some may move towards roles within healthcare that offer better work-life balance, or they may consider completely changing careers to find positions outside the healthcare sector [11].

Educational and professional development opportunities play a crucial role in shaping career pathways for nurses in Egypt. While there is a strong emphasis on education in the country, financial constraints and institutional limitations often hinder nurses’ ability to pursue further education or specialization. This lack of professional advancement opportunities drives many nurses to explore alternative career paths or even consider migrating to countries with more accessible and lucrative career options. This barrier to further education and specialization often leads nurses to reevaluate their career goals and seek opportunities where they can achieve personal and professional growth [26].

In addition to local factors, the global demand for healthcare professionals, including nurses, significantly influences career decisions. Many nurses in Egypt view opportunities to work abroad or in international organizations as an attractive option due to the promise of significantly higher salaries and better benefits. This global trend prompts Egyptian nurses to explore career pathways that could lead to international opportunities, where they can enhance their financial stability and career prospects. This external demand for healthcare workers also reinforces the perception that pursuing a career abroad is a viable and rewarding path for many nurses [27].

The role of financial motivations in nursing career decisions in Egypt can be attributed to a combination of economic realities, socio-cultural pressures, and global trends shaping the profession. Nurses are not only dealing with local financial challenges but are also responding to the global mobility of healthcare professionals, which further drives their career choices. The interplay of these factors highlights the complex decision-making process nurses face when choosing career pathways, with financial security often being a significant driver.

In relation to long-term career goals, many nursing graduates expressed interest in academic roles. This inclination may stem from the demanding nature of clinical practice, where nurses often experience challenging work conditions and less favourable work environments. Academic roles are seen as offering more stability, flexibility, and opportunities for intellectual growth. This finding aligns with previous studies that found a significant percentage of nursing students aspiring to become academics, citing the desire for a less stressful and more rewarding career path [6, 28]. Additionally, studies conducted in other countries, such as Turkey, also revealed that a notable proportion of nursing students aim to pursue academic careers, suggesting that this aspiration is shared across different cultural contexts [29].

The desire for academic roles may also be driven by the growing recognition of the need for well-educated nurse leaders who can contribute to the advancement of nursing practice and healthcare education. Nurses pursuing academic careers are in a unique position to influence the future of nursing by engaging in teaching, research, and policy development. This reflects a broader global trend in nursing, where higher education and academic positions are increasingly valued as essential for professional advancement and improving healthcare outcomes.

Thus, in the context of the Egyptian healthcare system, it is clear that multiple factors, including financial stability, working conditions, social expectations, and educational opportunities, play a significant role in shaping nursing graduates’ career choices. Nurses are navigating a complex landscape that involves balancing personal goals with the socio-economic realities of their profession. As the profession continues to evolve, understanding these factors is crucial for developing targeted strategies to support nurses in achieving their career aspirations and improving their job satisfaction.

Regarding nursing graduates’ tendency toward career shifts, over a quarter of them expressed consideration of such a change, indicating a significant proportion and potential signs of workforce instability and dissatisfaction within the nursing profession. This trend serves as a key indicator that systemic issues affecting job satisfaction and retention need to be addressed. High turnover rates, job dissatisfaction, and a desire to leave the profession are well-documented phenomena in recent studies [30, 31], further validating the concerns raised by the current study.

Financial growth (84.4%), work-life balance (74%), and a desire for a change (56%) were identified by nursing graduates as the leading factors influencing their consideration of career shifts. This aligns with findings from other studies, which highlight financial concerns, work-life balance, and the desire for professional change as prominent drivers for career shifts or leaving the profession [32–34]. The financial aspect is particularly relevant given the non-competitive salaries in the healthcare sector, which do not match the rising cost of living. Work-life balance issues, including long hours, overtime, and limited time off, also contribute to nurses’ dissatisfaction. Meanwhile, the desire for a change can reflect a wish for new challenges and personal growth opportunities.

Regarding the challenges anticipated in pursuing their chosen career pathways, nursing graduates reported several obstacles. The most commonly cited challenges were lack of financial resources (60.9%), limited time for further education (53%), and job market competitiveness (31.4%). These factors, combined with financial concerns, may discourage nursing graduates from pursuing career shifts due to apprehensions about change and the uncertainty of delayed financial rewards in new fields. This suggests that despite the desire for career change, practical barriers remain a significant concern for many nursing graduates.

In terms of the fields or specializations considered for a career shift, nursing graduates expressed interest in various sectors. These included marketing management (49%), real estate development (36%), freelancing (27.9%), social media work (27.4%), and trading (26.0%). These results are in line with studies on German and European nurses, which have identified similar fields as attractive career options for those considering leaving the nursing profession [35, 36]. The appeal of these fields lies in their perceived opportunities for career growth, financial rewards, and flexibility. The rise of digital technologies and the expansion of global markets have likely increased the accessibility and attractiveness of these sectors to nurses seeking new professional challenges outside traditional nursing roles.

Several confounding variables can influence career perceptions and shift tendencies among nursing graduates, and these need to be considered when evaluating career pathway decisions. Educational qualifications are a significant factor in shaping career satisfaction and the likelihood of career shifts. Nurses with higher levels of education, such as bachelor’s or master’s degrees, generally report higher career satisfaction and greater stability, making them less likely to consider a career change [27]. This suggests that investing in education could potentially help mitigate some of the factors that contribute to career dissatisfaction and shifts.

Age and professional experience also play a role in career decisions. Older nurses and those with more years of experience tend to feel more secure in their roles and are less likely to pursue career shifts. This is supported by research indicating that experienced nurses report higher levels of job satisfaction and a stronger sense of professional identity, which reduces their likelihood of leaving the profession [37]. Conversely, younger nurses with less experience may be more open to change and explore different career paths.

This is often due to physical exhaustion, burnout, and dissatisfaction with working conditions in clinical roles. In contrast, younger nurses tend to have more flexibility in their career decisions and are more open to exploring different pathways, such as roles in healthcare administration or academia. Interventions targeted at older nurses, such as opportunities for less physically demanding roles or mentorship programs, can improve retention and job satisfaction. For younger nurses, providing clear professional development pathways and continuous education can enhance career commitment and reduce the likelihood of early career shifts [38].

Job satisfaction is another crucial factor influencing career decisions. Poor working conditions, including high nurse-to-patient ratios, inadequate staffing, and limited organizational support, can lead to burnout and dissatisfaction. Studies have consistently found that nurses experiencing poor job satisfaction are more likely to consider leaving their roles [39]. As such, improving workplace conditions is essential to reducing turnover intentions and preventing unnecessary career shifts.

Socioeconomic factors, such as economic pressures and the cost of living, are particularly relevant in countries like Egypt. Nurses may prioritize financial security, which can drive career shifts if they perceive limited financial rewards in their current roles. Additionally, cultural expectations, particularly concerning gender roles and family responsibilities, can influence career decisions, particularly for women who may feel societal pressure to balance work with familial duties. These cultural and socioeconomic factors highlight the complex decision-making process that nursing graduates face when considering career shifts.

In conclusion, the tendency toward career shifts among nursing graduates is driven by a combination of financial, personal, and systemic factors. Financial growth, work-life balance, and a desire for change are significant motivators, while challenges such as lack of financial resources, time constraints for further education, and market competitiveness remain obstacles. The fields considered for career shifts reflect the evolving job market and nurses’ search for opportunities that offer better financial prospects, flexibility, and growth. Addressing these issues through policy changes, improved working conditions, and better educational opportunities is essential for retaining skilled nursing professionals and ensuring the stability of the healthcare workforce.

Additionally, the global demand for healthcare professionals has created significant opportunities for nurses to migrate abroad for better-paying jobs and career advancement. This potential for migration has influenced the perceptions of nursing graduates in Egypt, with many viewing foreign job opportunities as more attractive than staying within the local healthcare system. Recent studies have highlighted the impact of global workforce mobility on nurses’ career choices. For example, research by [40] found that the perception of better opportunities abroad was a major factor in nurses’ decisions to leave their home countries. Such opportunities are often seen as offering higher salaries, better working conditions, and greater professional growth, making them appealing to nurses facing challenges within the domestic healthcare system.

When examining the impact of gender on career pathway perceptions and career shift tendencies, the current study found no statistically significant difference between male and female nurses. This suggests that gender does not play a significant role in shaping career decisions or the likelihood of career shifts among nursing graduates in Egypt. This finding contrasts with some previous studies, where gender-related factors, such as societal expectations and familial responsibilities, were found to influence career choices [35]. Regarding age, a noticeable difference emerged between younger and older nurses. Younger nurses, who are often more enthusiastic and passionate about their careers, were more likely to express a desire for professional development and workplace innovation. Older nurses, on the other hand, exhibited a greater intention to leave the profession, possibly due to burnout or dissatisfaction with career progression [36].

The current study also explored the role of educational qualifications in shaping career pathway perceptions. A statistically significant relationship was found between nurses’ degrees, their current employment status, and their career pathway intentions. Nurses with higher qualifications, such as a bachelor’s or master’s degree, were more likely to express a strong motivation toward their profession compared to those with less education [41]. Research by Watts et al. [27] further supports this, showing that certified nurses were less likely to leave the profession compared to their non-certified counterparts. This suggests that higher educational qualifications may lead to greater job satisfaction, career stability, and lower intentions to shift careers.

Furthermore, the study revealed a significant relationship between demographic factors, such as age, years since graduation, and years of experience, with career pathway perceptions and career shift tendencies. Older nurses and those with more years of experience were less likely to consider a career shift, reflecting a negative correlation with career change tendencies (*p* < 0.001). This finding aligns with previous research, which suggests that experienced nurses tend to experience higher job satisfaction, develop a stronger professional identity, and exhibit more career stability [35]. Older nurses are also less inclined to shift careers due to concerns about job security, retirement, and the difficulties associated with starting anew in a different field [36].

These insights underscore the importance of considering demographic factors when developing strategies to support career pathways and workforce retention in healthcare settings. Understanding the unique needs and motivations of different nurse cohorts—based on their age, qualifications, and experience—can help tailor interventions that address specific challenges and improve job satisfaction.

The findings of this study also emphasize the importance of addressing factors that contribute to career shift tendencies, such as financial pressures, work-life balance, and the desire for professional change. To mitigate these issues and enhance career satisfaction, the study suggests implementing targeted interventions that focus on improving workplace conditions, promoting work-life balance, and providing ample opportunities for career advancement and professional development. By addressing these key factors, healthcare organizations can foster a more satisfied and committed nursing workforce, ultimately contributing to the quality of patient care.

Additionally, the study recommends proactive measures to address the factors driving intentions to leave the profession, such as improving work environments, fostering intergenerational collaboration, and offering mentorship programs. These interventions can help retain nursing professionals and create a more cohesive, resilient workforce capable of meeting the challenges of modern healthcare delivery.

To enhance career retention and satisfaction among nursing graduates in Egypt, targeted interventions are needed, especially to address work-life balance and mentorship. Mentorship programs could be implemented by pairing experienced nurses with recent graduates to provide career guidance, emotional support, and professional development. These programs would help young nurses navigate the early stages of their careers, improve job satisfaction, and reduce the likelihood of career shifts. Mentorship could be particularly effective in retaining talent and fostering professional growth, especially for those feeling uncertain about their career pathways.

Work-life balance initiatives are also crucial, particularly for female nurses in Egypt who often juggle professional and familial responsibilities. Healthcare institutions could introduce flexible work schedules, shift rotations, and childcare support to alleviate the pressures on nurses. Such policies would not only improve nurses’ well-being but also enhance their job satisfaction, potentially reducing turnover and encouraging retention. These changes would help nurses manage their personal and professional responsibilities more effectively, contributing to long-term career stability in the Egyptian healthcare sector.

In conclusion, this study provides valuable insights into the career perceptions and shift tendencies of Egyptian nursing graduates. It highlights positive perceptions regarding their career pathways, over a quarter of them were contemplating career shifts, indicating potential workforce instability and dissatisfaction. The study also highlighted that financial challenges, low salaries, limited career advancement, and inadequate resources which could influence their career choices, including financial concerns, educational qualifications, and workplace conditions, as well as the role of demographic factors such as age and experience. To support the retention and career satisfaction of nursing professionals, it is essential to implement targeted strategies that address these concerns and promote professional growth. Future research with larger sample sizes would further deepen our understanding of these issues and guide the development of more effective workforce retention strategies in healthcare settings.

### Limitations

The large population of nursing graduates in Egypt, with an estimated 218,000 graduates in the field (according to the Central Agency for Public Mobilization and Statistics, 2021), made it challenging to obtain a truly representative sample that could reflect the diverse demographics, career stages, and professional experiences within this vast group. The study employed a convenience sampling method, which, while accessible and cost-effective, may introduce selection bias and limit the generalizability of the results. This approach could lead to an over- or under-representation of specific group.

To mitigate this limitation, we took several steps to diversify our sample and reach a broader, more representative audience of nursing graduates, we conducted wide-reaching recruitment through diverse sources, including professional nursing organizations, hospitals, universities, and online platforms, ensuring access to various subgroups within the population. We also used multiple communication channels, such as email, phone, and social media, to reach participants across different locations and technological access levels. Additionally, we actively monitored demographics during data collection to target underrepresented groups, applied statistical weighting to correct sampling imbalances, and emphasized the study’s relevance for all career stages to encourage broad participation. These steps helped enhance the representativeness of our sample, providing a balanced perspective on nursing career aspirations and shift tendencies across Egypt.

## Conclusion

The present study delved into career pathway perceptions of Egyptian nursing graduates, revealing a notable tendency to consider career shifts, driven by factors such as financial growth, work-life balance, and the desire for new opportunities. The most popular career alternatives identified were in fields such as marketing management, real estate development, freelancing, social media, and trading. While the majority of nursing graduates expressed positive perceptions regarding their career pathways, over a quarter of them were contemplating career shifts, indicating potential workforce instability and dissatisfaction. The study also highlighted that financial challenges, low salaries, limited career advancement, and inadequate resources in the healthcare system were significant drivers behind these career considerations. Economic factors, such as the rising cost of living and inflation, further emphasized the role of financial security in career decision-making. Additionally, the study found that older nurses were more likely to consider leaving the profession, while there was a significant relationship between nursing graduates’ education level, current employment status, and their career perceptions. Moving forward, future research should focus on evaluating the effectiveness of interventions like mentorship programs, work-life balance initiatives, and career development opportunities in enhancing nurse retention and job satisfaction. Studies should also explore how professional development opportunities, such as continuing education and specialization, impact long-term career trajectories. Further research could examine how cultural and socio-economic factors, such as gender and age, influence career decisions, and assess the impact of international migration trends on the nursing workforce. These areas of exploration will help develop targeted strategies to support a stable and satisfied nursing workforce, ensuring both high-quality patient care and sustainable healthcare systems.

## Supplementary Information

Supplementary Material 1.

## Data Availability

The datasets generated and analyzed during the current study are available from the corresponding author. https://docs.google.com/forms/d/e/1FAIpQLSfz4TQIzpkWGwRIYC1T-P9anVvdogUvT73nMwb-NY5XdPzEqw/viewform This is the link of google form used to collect data from study subjects and data are available on the google drive of corresponding author (Mohamed Hashem Kotp).
