# Supplementary material for: Nursing graduates’ perceived future career pathway and career shift tendency in Egypt: a cross sectional study
Source: BMC Nurs. 2025 Feb 18;24:190. doi: 10.1186/s12912-025-02709-6 (PMC11837679; doi:10.1186/s12912-025-02709-6)
Supplement: Supplementary file 1 — Supplementary Material 1. [file 12912_2025_2709_MOESM1_ESM.pdf]

Nursing graduates' perceived future career pathway and career shift tendency in Egypt

This questionnaire designed to understand and evaluate the opinions of nursing graduates regarding their perceived future career pathways and tendencies towards career shifts.

We would like to inform you that any information you provide will be treated with strict confidentiality. Your responses will be anonymized, and no personally identifiable information will be disclosed in any reports or publications arising from this study.

Please answer the following questions honestly, as your participation will contribute to enhancing our understanding of this important topic.

I agree participating in this study and accept publication of data generated from this study.

- Yes ( )
- No ( )

Part A: Personal characteristics:

Gender

- Male ( )
- Female ( )

Age

- Less than 25 years. ( )
- 25-30 years ( )
- 31-35 years ( )
- 36-40 years ( )
- More than 40 years ( )

Highest Nursing Degree Earned

- Diplome ( )
- Associate degree ( )
- Bachelor of Science in Nursing (BSN) ( )
- Master of Science in Nursing (MSN) ( )
- Diploma of Nursing Practice (DNP). ( )
- Nursing PhD. ( )

Years Since Graduation (Experience)

- 1-5 years. ( )
- 6-10 years. ( )
- 11-15 years. ( )
- Over 15 years. ( )

Current Employment Status

- Employed as a bed side nurse. ( )
- Employed as nurse manager. ( )
- Healthcare quality nurse ( )
- Employed in a non-nursing role. ( )
- Academic educational role. ( )
- Case manager. ( )
- Free lancer nurse. ( )
- Nursing science researcher. ( )
- Nursing informatics specialist. ( )

Current Work Setting

- Governmental Hospital. ( )
- Private sector hospital. ( )
- Primary healthcare facility. ( )
- University faculty. ( )
- Own business nursing facility. ( )

Part B:

Future Career Pathway perception

Item	Agree	Neutral	Disagree
**Immediate career goals**			
I Plan for advancement in the current nursing role (e.g., managerial roles)			
I Pursue a specialized nursing certification (e.g., Critical Care, Oncology)			
I Pursue advanced nursing education (e.g., MSN, DNP)			
I Plan for transition to a different nursing specialty			
Plan for transition to a non-nursing healthcare role			
**Motivators to explore current pathway**			
Passion for the field.			
Opportunities for advancement.			
Desire to make a difference.			
Financial prospects.			
**Challenges or obstacles anticipated in pursuing chosen career pathway**			
Lack of financial resources			
Limited time for additional education			
Job market competitiveness			
Family commitments			
**Long-term career goals as a nursing graduate**			
Nursing leadership/management roles			
Nurse educator/teaching roles			
Academic roles.			
Nursing informatics role.			
Healthcare quality role.			
Nurse researcher/clinical research			
Advanced practice nursing (e.g., Nurse Practitioner)			
Transition to non-healthcare career			

Part C: Career shift tendency.

Item	Agree	Neutral	Disagree
**Considering career shift**			
I consider shifting my career within months			
**Factors influencing consideration of a career shift.**			
Desire for a Change			
Better Career Opportunities			
Work-Life Balance			
Financial Growth			
International Experience			
**Challenges or obstacles anticipated in pursuing chosen career pathway.**			
Lack of financial resources			
Limited time for additional education			
Job market competitiveness			
Family commitments			
**Field or specialization considered for a career shift.**			
Software Developer			
Social media work			
Telesales			
Marketing management			
Freelancing			
Real Estate Development			
Tourism			
Trading			
